# microRNA Profile Associated with Positive Lymph Node Metastasis in Early-Stage Cervical Cancer

**DOI:** 10.3390/curroncol29010023

**Published:** 2022-01-08

**Authors:** Salim Abraham Barquet-Muñoz, Abraham Pedroza-Torres, Carlos Perez-Plasencia, Sarita Montaño, Lenny Gallardo-Alvarado, Delia Pérez-Montiel, Luis Alonso Herrera-Montalvo, David Cantú-de León

**Affiliations:** 1 Programa de Maestría y Doctorado en Ciencias Médicas, Odontológicas y de la Salud, UNAM, Mexico City 14080, Mexico; sbarquetm@incan.edu.mx; 2 Cátedra CONACYT-Basic Research Division, Instituto Nacional de Cancerología, Mexico City 14080, Mexico; apedrozato@conacyt.mx; 3 Genomics Laboratory, Basic Research Division, Instituto Nacional de Cancerología, Mexico City 14080, Mexico; carlos.pplas@unam.mx; 4 Bioinformatics Laboratory, Biological Chemistry Sciences Department, Universidad Autónoma de Sinaloa (FCQB-UAS), Culiacan 80040, Mexico; mmontano@uas.edu.mx; 5 Clinical Research Subdivision, Basic Research, Instituto Nacional de Cancerología, Mexico City 14080, Mexico; lgallardoa@incan.edu.mx; 6 Department of Pathology, Instituto Nacional de Cancerología, Mexico City 14080, Mexico; mperezm@incan.edu.mx; 7 Cancer Biomedical Research Unit, Instituto Nacional de Cancerología and Instituto Nacional de Medicina Genómica, Mexico City 14080, Mexico; herreram@biomedicas.unam.mx; 8 Division of Research, Instituto Nacional de Cancerología, Av. San Fernando #22, Mexico City 14080, Mexico

**Keywords:** miRNAs, cervical carcinoma, lymph node metastasis

## Abstract

Lymph node metastasis (LNM) is an important prognostic factor in cervical cancer (CC). In early stages, the risk of LNM is approximately 3.7 to 21.7%, and the 5-year overall survival decreases from 80% to 53% when metastatic disease is identified in the lymph nodes. Few reports have analyzed the relationship between miRNA expression and the presence of LNM. The aim of this study was to identify a subset of miRNAs related to LNM in early-stage CC patients. Formalin-fixed paraffin-embedded tissue blocks were collected from patients with early-stage CC treated by radical hysterectomy with lymphadenectomy. We analyzed samples from two groups of patients—one group with LNM and the other without LNM. Global miRNA expression was identified by microarray analysis, and cluster analysis was used to determine a subset of miRNAs associated with LNM. Microarray expression profiling identified a subset of 36 differentially expressed miRNAs in the two groups (fold change (FC) ≥ 1.5 and *p* < 0.01). We validated the expression of seven miRNAs; miR-487b, miR-29b-2-5p, and miR-195 were underexpressed, and miR-92b-5p, miR-483-5p, miR-4534, and miR-548ac were overexpressed according to the microarray experiments. This signature exhibited prognostic value for identifying early-stage CC patients with LNM. These findings may help detect LNM that cannot be observed in imaging studies.

## 1. Introduction

Cervical carcinoma (CC) is one of the most common cancers in women from low-income and middle-income countries; in Mexico, CC is the second most common tumor and has a high mortality rate [1]. The most important factors affecting survival of patients with early-stage CC (International Federation of Gynecology and Obstetrics (FIGO) stage IA–IB1) are the clinical stage, tumor size, depth of tumor invasion into the stroma, lymphovascular space invasion (LVSI), and presence of lymph node metastasis (LNM). The primary treatment is radical hysterectomy (RH) with bilateral pelvic lymphadenectomy (BPL) [2].

In early stages, the risk of LNM is approximately 3.7 to 21.7%, and the 5-year overall survival (OS) decreases from 80% to 53% when metastatic disease is identified in the lymph nodes (LNM+) [3,4,5,6]. Therefore, the FIGO updated their staging system in 2018 by incorporating nodal status into stage III disease. The standard technique to identify positive lymph nodes is based on advanced imaging methods such magnetic resonance imaging (MRI), computed tomography (CT), or positron emission tomography–CT (PET)–CT. The accuracy of each of these modalities ranges from 70 to 85%, and the lymph nodes must reach a minimum size to be identified by imaging studies. Microscopic disease cannot be detected by noninvasive methods [7,8,9,10].

MicroRNAs (miRNAs) are single-stranded RNAs comprising approximately 21–23 nucleotides and are essential in a wide variety of physiological and pathological processes, including development, differentiation, metabolism, immunity, cell cycling, proliferation, apoptosis, cell identity, and stem cell maintenance. They regulate gene expression by inhibiting posttranscriptional events and, in some cases, they induce the degradation of their target messenger RNA [11,12,13]. In cancer, miRNAs can function as oncogenes and/or tumor suppressor genes depending on the function of their target genes. Abnormal expression of miRNAs is linked to several features of cancer biology, including proliferation, differentiation, apoptosis, migration, invasion, and metastatic angiogenesis [14,15,16,17].

Numerous studies have demonstrated the role of miRNAs in the development and prognosis of CC [18,19,20,21]. Some of the miRNAs identified in CC include miR-23b, miR-143, let-7b, let-7c, miR-196b, miR-9, miR-127, miR-133a, miR-133b, and miR-145 [13,22]. Moreover, some studies have shown that miRNAs act as prognostic markers in CC [13,23,24,25]. As previously mentioned, MRI, CT, and PET–CT scans cannot identify all patients with early-stage CC and LNM, and the use of miRNAs as molecular markers for the LNM+ status in the early stages of CC remains poorly studied. The aim of this study was to identify a subset of miRNAs related to positive LNM in early-stage CC patients.

## 2. Materials and Methods

### 2.1. Patients and Tumor Samples

Formalin-fixed paraffin-embedded (FFPE) tissue blocks from early-stage CC patients treated with RH and BPL who were diagnosed between January 2006 and December 2013 at the Instituto Nacional de Cancerología (Mexico City) were reviewed. The exclusion criteria were patients with 2 primary tumors, primary treatments other than RH, or poor quality of the tissue in the FFPE blocks. The FFPE blocks were stained with hematoxylin and eosin (H&E) to identify tumor regions and verify a minimum presence of 80% of tumor cells. Subsequently, total RNA was extracted from 5 sections (10 µm) from the selected FFPE blocks. For the analysis, LNM+ patients were paired with a similar number of patients without LNM (LNM-) matched by age, tumor size, and the presence of LVSI.

### 2.2. RNA Extraction

Total RNA was extracted using an miRNeasy FFPE Kit (QIAGEN) according to the manufacturer’s instructions. First, 5 sections (10 µm) were deparaffinized using 320 µL of deparaffinization solution and incubated at 56 °C for 3 min. Subsequently, the samples were treated with 10 µL of Proteinase K and incubated at 56 °C for 15 min and at 80 °C for 15 min. Then, the samples were treated with 500 µL of RBC buffer to adjust the binding conditions. Next, 500 µL of RPE buffer was added to the samples, and the mixture was transferred to a RNeasy MinElute spin column. Finally, total RNA was eluted in 50 µL of RNase-free water.

To verify the usefulness of the samples, RNU6B was amplified by RT-qPCR using TaqMan miRNA probes and the TaqMan MicroRNA Reverse Transcription Kit (Applied Biosystems, Waltham, MA, USA) according to the manufacturer’s specifications. Samples for which RNU6B amplification was possible and that showed an adequate amount of total RNA (>50 ng/µL) were used to identify their global miRNA expression profile.

### 2.3. Global miRNA Expression Profiles

Global miRNA profiles were identified using a GeneChip miRNA 3.0 Array (Affymetrix, Cat. 902018) following the manufacturer’s instructions. The GeneChip miRNA 3.0 Array contains 19,724 probe sets and can quantify 1733 human mature miRNAs (miRBase v17). Initially, 500 ng of total RNA was labeled using a FlashTag Biotin RNA Labeling Kit (Affymetrix, Santa Clara, CA, USA). Then, a poly(A) tailing reaction was performed at 37 °C for 15 min (1X reaction buffer, 750 µL of MgCl_2_ (25 mM), 500 µL of adenosine triphosphate (ATP), and 500 µL of propyl aminopeptidase (PAP) enzyme). FlashTag ligation was immediately conducted at room temperature for 30 min (2 mL of 5X FlashTag Ligation Mix Biotin and 1 mL of T4 DNA ligase), and 1.2 mL of stop solution was added to stop the reaction. Finally, the microarray was hybridized and washed using an Affymetrix Fluidics Station 450 and scanned with the Affymetrix GeneChip Scanner 3000. After processing the images, the raw data were obtained. Then, background correction and normalization were performed by the quantiles method using the robust multiarray average (RMA) tool in the affy package in R (3.5.1 v). To obtain the miRNA profile, the processed samples were divided into 2 groups, LNM+ samples and LNM− samples, using the Limma package (Linear Models for Microarray Data) in R (3.5.1 v). Differentially expressed miRNAs were identified using a cutoff value of *p* < 0.01 and a fold change (FC) of 1.5. Finally, we constructed a heatmap with the differentially expressed miRNAs in the two groups of samples using the heatmap.2 function in R (3.5.1 v) and the nonsquared Euclidean distances method (Ward.D2) [26].

### 2.4. Validation of the miRNA Profiles by RT-qPCR

Ten miRNAs were selected to validate the data obtained through the microarray experiments in the same cohort of patients (*n* = 20, 10 LNM+ samples and 10 LNM− samples) using miRNA-specific stem-loop primers and a TaqMan MicroRNA Reverse Transcription Kit. First, cDNA was synthesized using specific primers for each miRNA according to the manufacturer’s specifications. Subsequently, the quantification reaction consisted of 10 μL of 2X TaqMan Fast Universal PCR Master Mix No AmpErase UNG, 1 μL of 0.2 μM TaqMan probe, and 1.33 μL of cDNA. All reactions were performed in triplicate using a GeneAmp PCR system 9700 thermal cycler (Applied Biosystems) with the following thermocycling program: 16 °C for 30 min, 42 °C for 30 min, and 85 °C for 5 min. RNU6B (assay ID: 001093) was used as an endogenous control to normalize the miRNA expression level. The mean Ct values in each qPCR were used to calculate miRNA relative expression. Finally, 2^−ΔΔCt^ values were plotted [27].

### 2.5. Prediction of miRNA Target Genes and Pathways

To identify which genes and signaling pathways were regulated by the validated miRNA signature associated with LNM+ samples, we used the miRWalk 3.0 and DAVID 6.8 bioinformatics tools [28,29]. miRWalk 3.0 is a bioinformatics tool that allows the prediction of target genes through the miRNA–target gene interaction using the TarPmiR algorithm [30]. This approach allowed us to predict interactions between 5′-untranslated region (UTR), coding sequence (CDS), and 3′-UTR and the seed region of the miRNA candidate. In addition, miRWalk 3.0 uses the datasets of 2 prediction platforms (TargetScan and miRDB) and the experimentally validated interaction information from miRTarBase. First, we selected miRNAs with expression validated by RT-qPCR and those that showed clinical significance to search for their target genes using the miRWalk 3.0 tool. Then, with the list of genes obtained, we performed Gene Ontology (GO) enrichment and Kyoto Encyclopedia of Genes and Genomes (KEGG) analyses to determine the functions of associated genes and pathways [31,32]. Finally, the interaction network between target genes and selected miRNAs was visualized with Cytoscape and CyTargetLinker bioinformatic tools [33,34].

### 2.6. Statistical Analysis

Quantitative data are expressed as the mean ± standard deviation (SD). miRNA expression levels were compared between the 2 groups by an unpaired t test. The chi-square test or Fisher’s exact test was performed to assess the relationships between miRNA expression and clinical features. To determine the clinical correlation between the LNM+ status and the miRNA profile, logistic regression was performed between the expression values of miRNAs and lymph node status to calculate the odds ratio (OR). A p value less than 0.05 was considered statistically significant. Statistical analyses were performed using SPSS, version 23 (IBM Corp., Armonk, NY, USA). This study was approved by the local institutional review boards, with approval reference (INCAN/CI/248/15).

## 3. Results

### 3.1. Patients and Samples

A total of 25 samples were selected. Twelve samples were selected from LNM+ patients, and 13 samples were selected from LNM− patients. The clinical and histopathological characteristics are summarized in Table 1. A significant difference was found for the type of adjuvant treatment (*p* = 0.02) and depth of invasion (*p* = 0.04).

### 3.2. miRNA Profile Associated with LNM+ Patients

**Figure 1:**
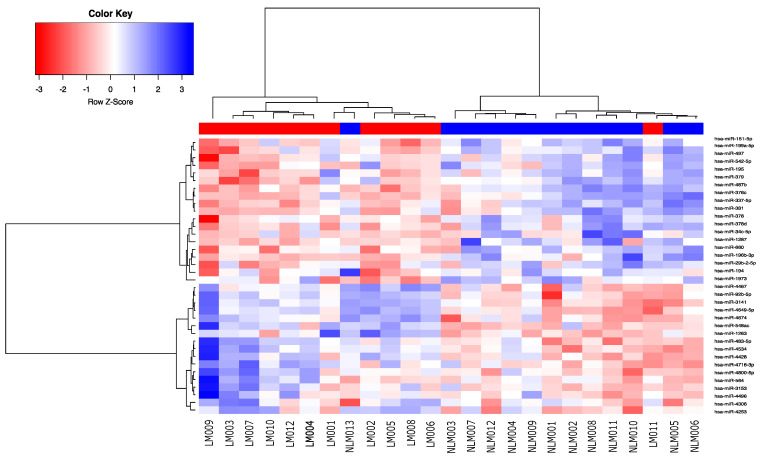
Heatmap of the supervised hierarchical clustering analysis of the 36 miRNAs expressed in the lymph node-positive group and the control group. The 12 samples in red exhibited lymph node metastasis (LNM), and the 13 samples in blue did not exhibit LNM.

Microarray expression profiling identified a subset of 36 differentially expressed miRNAs between the 2 groups (FC ≥ 1.5 and *p* < 0.01). A heatmap of the differentially expressed miRNAs showed that the samples were grouped with respect to lymph node status (Figure 1 and Supplementary File S1). Among the identified miRNAs, 17 were overexpressed, and 19 were underexpressed. Among the main dysregulated miRNAs, miR-487b (FC = −3.2, *p* = 0.0003), miR-194 (FC = −2.8, *p* = 0.006), miR-34c-5p (FC = −2.46, *p* = 0.007), miR-29b-2-5p (FC = −2.3, *p* = 0.007), and miR-195 (FC = −2.07, *p* = 0.001) were underexpressed, while miR-548ac (FC = 2.74, *p* = 0.0003), miR-4534 (FC = 2.47, *p* = 0.001), miR-483-5p (FC = 2.21, *p* = 0.002), miR-564 (FC = 2.01, *p* = 0.006), and miR-92b-5p (FC = 1.82, *p* = 0.005) were overexpressed. This molecular signature correctly classified 91.6% (11/12) of the LNM+ samples and 92.3% (12/13) of the LNM− samples.

### 3.3. Validation of miRNAs by RT-qPCR

**Figure 2:**
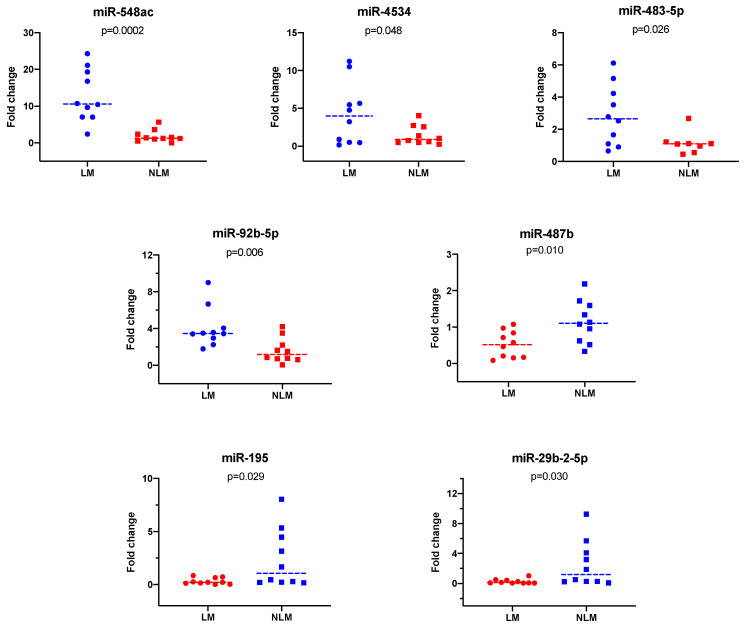
The seven miRNAs validated by RT-qPCR experiments. We were able to validate the expression of 7 of the 10 selected miRNAs by RT-qPCR experiments. miR-548ac, miR-4534, miR-483-5p, and miR-92b-5p were overexpressed, whereas miR-487b, miR-195, and miR-29b-2-5p were underexpressed. LNM+: patients with lymph node metastasis, LNM−: patients without lymph node metastasis. miRNAs underexpressed in the microarray that were subjected to validation. The miRNAs miR-487b, miR-29b, and miR-195 were validated by RT-qPCR. LM: patients with lymph node metastasis, NLM: patients without lymph node metastasis.

To validate the data obtained through the microarray experiments, we selected 10 miRNAs based on their expression levels and *p* values. We were able to validate the expression of 7 of the 10 selected miRNAs by RT-qPCR experiments (Figure 2); miR-487b, miR-29b-2-5p, and miR-195 were underexpressed, and miR-92b-5p, miR-483-5p, miR-4534, and miR-548ac were overexpressed according to the microarray experiments. The remaining three miRNAs showed positive correlations in the microarray experiments but did not show statistically significant differences (miR-194, miR-34c-5p, and miR-564).

**Table 1:** Clinical and histopathological characteristics of the patients with cervical carcinoma analyzed in this study.

N (%)	With Lymph Node Involvement 12 (48)	Without Lymph Node Involvement 13 (52)	*p*
Age +	54.92 ± 8.89	54.85 ± 10.80	0.98
Clinical Stage ^ Ia2 Ib1 IIa1	0 (0.0) 12 (100.0) 0 (0.0)	1 (7.7) 10 (76.9) 2 (15.4)	
BMI +	28.82 ± 2.69	30.36 ± 4.23	0.34
BMI2 ^ ≤25 25.1–30 >30	3 (25.00) 4 (33.33) 5 (41.67)	3 (23.08) 2 (15.38) 8 (61.54)	0.67
Type of adjuvant treatment ^ None EBR or BT CT/EBR+BT	0 (0.00) 4 (33.33) 8 (66.67)	4 (30.77) 7 (53.84) 2 (15.38)	0.02
Type of recurrence ^ None Local Regional Distant	10 (83.33) 1 (8.33) 0 (0.0) 1 (8.33)	13 (100) 0 (0.0) 0 (0.0) 0 (0.0)	0.22
Histology ^ SCC Adenocarcinoma ASCC	8 (66.67) 3 (25.00) 1 (8.33)	10 (76.92) 1 (7.69) 2 (15.38)	0.57
Grade ^ 1 2 3	0 (0.0) 7 (58.33) 5 (41.67)	1 (7.69) 7 (53.85) 5 (38.46)	0.90
LVSI ^	11 (91.67)	10 (76.92)	0.33
Invasion depth in mm *	15 (10.5–17)	10 (7–11)	0.04
Thirds ^ 1/3 2/3 3/3	1 (8.33) 2 (16.67) 9 (75.00)	3 (23.08) 2 (15.38) 8 (61.54)	0.83
TZ in mm +	30.75 ± 9.55	25.23 ± 12.07	0.22
Positive margins ^	3 (25.00)	3 (23.08)	0.90
Parametrial involvement ^	4 (33.33)	1 (7.69)	0.16
Lymph nodes count ^	20 (17.5–29)	19 (12–28)	0.46

Abbreviations: BMI: body mass index; EBR: external beam radiotherapy; BT: brachytherapy; CT/EBRT concurrent chemotherapy and external beam radiotherapy; SCC: squamous cell carcinoma; ASCC: adenosquamous cell carcinoma; LVSI: lymphovascular space invasion; TZ: tumor size; + mean ± standard deviation; ^ absolute value (%); * median (interquartile range).

### 3.4. Identification of miRNA Target Genes and Signaling Pathways

To identify gene targets and signaling pathways associated with the LNM+ status, we selected the seven miRNAs that were validated by RT-qPCR and showed clinical significance.

**Figure 3:**
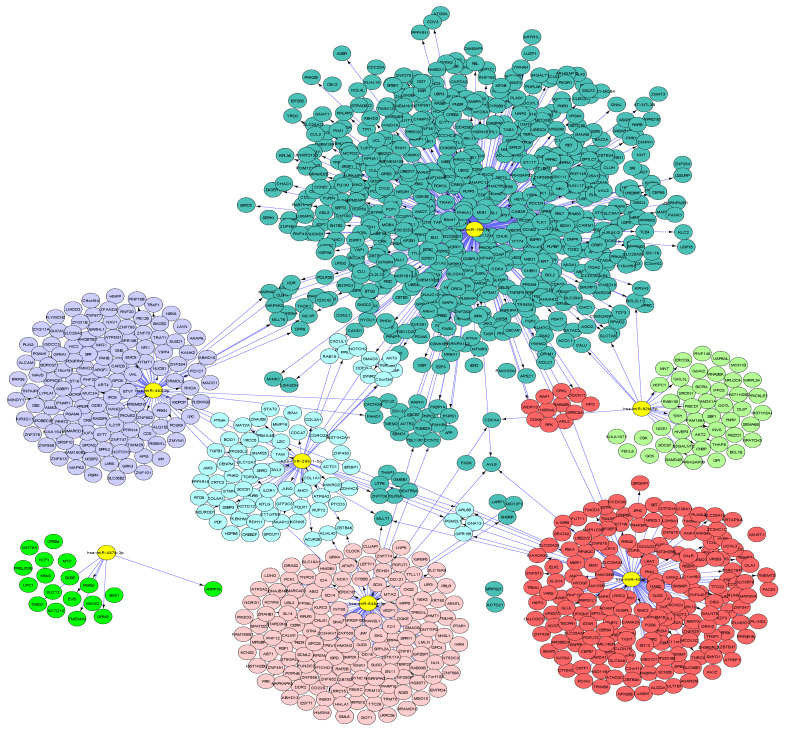
Interaction networks between target genes and the seven-miRNA signature. Target genes and the seven-miRNA signature were visualized with Cytoscape and CyTargetLinker. miRNAs are marked in yellow, while target genes are color-coded according to their regulatory miRNAs.

After bioinformatic analysis using miRWalk 3.0, we found 468 target genes (putative and validated) for this 7-miRNA molecular signature. Next, we performed KEGG and GO functional enrichment analyses using DAVID 6.8. The biological process terms were markedly enriched for regulation of transcription, regulation of cell migration, protein ubiquitination, epithelial cell–cell adhesion, and regulation of NF-kappa B transcription (Table 2). For the GO cellular component terms, the target genes were concentrated in the nucleus, cytosol, extracellular exosome, membrane, and rough endoplasmic reticulum, among others. The molecular function terms were enriched for protein binding, transcription factor binding, and beta-catenin binding. The KEGG pathways were predominantly enriched for cancer pathways, viral carcinogenesis, and the MAPK, ErbB, and GnRH signaling pathways (Supplementary File S1). In addition, interaction networks between target genes and the 7-miRNA signature were visualized with Cytoscape and CyTargetLinker (Figure 3 and Supplementary File S1) [33,35].

**Table 2:** Biological processes enriched in target genes.

GO Term	Count	*p* Value	Genes
Transcription from RNA polymerase II promoter	19	9.3 × 10^−5^	DDX21, FOSL1, GABPA, IKZF3, MAFK, MNT, MLLT1, PLAGL2, CLOCK, CCNT1, CCNT2, FOXC1, FOXJ2, GTF2H5, HIVEP3, MBD1, NFIC, NFIX, SRF.
Regulation of transcription, DNA-templated	35	6.1 × 10^−4^	KANK1, LRRFIP2, MACC1, MLLT1, MLLT6, POM121C, SMAD3, THAP1, TMEM189-UBE2V1, BZW1, CALR, CLOCK, CPNE1, FOXC1, GTF2H5, KAT6A, KDM2A, MKX, PTPN14, RNF20, SRSF10, TCF3, UBE2V1, VHL, ZBTB10, ZBTB34, ZBTB8A, ZNF276, ZNF391, ZNF426, ZNF429, ZNF585B, ZNF662, ZNF747, ZNF813.
Negative regulation of cell migration	7	1.3 × 10^−3^	BCL2, KANK1, ARHGDIA, SRGAP1, RNF20, SRF, VCL.
Protein ubiquitination	13	2.1 × 10^−3^	DCAF17, CDC42, CUL3, CAND1, MIB1, PARK2, RNF138, RNF168, SOCS5, SOCS7, UBE2Q1, VHL, ZYG11B.
Positive regulation of transcription from RNA polymerase II promoter	24	3.1 × 10^−3^	FOSL1, GABPA, IKZF3, MAFK, PAGR1, PLAGL2, SMAD3, APP, CLOCK, CCNT1, CCNT2, FGF2, FOXC1, FOXJ2, MAVS, MAPK3, NFIC, NFIX, PARK2, RPRD1B, SRF, TCF3, TBL1XR1, TGFB1.
Nucleotide-binding oligomerization domain-containing signaling pathway	4	3.7 × 10^−3^	CYLD, TAB3, TMEM189-UBE2V1, UBE2V1
Epithelial cell–cell adhesion	3	5.4 × 10^−3^	CDC42, SRF, VCL.
Positive regulation of transcription, DNA-templated	15	5.9 × 10^−3^	SMAD3, TMEM189-UBE2V1, CLOCK, FGF2, FOXC1, FOXJ2, HIVEP3, KAT6A, MAPK3, RNF20, TCF3, TBL1XR1, TGFB1, UBE2V1, VHL.
Positive regulation of NF-kappa B transcription factor activity	8	7.6 × 10^−3^	KRAS, TAB3, TMEM189-UBE2V1, CAMK2A, CLOCK, TGFB1, UBE2V1.
Transcription, DNA-templated	38	9.3 × 10^−5^	HIC2, KANK1, MACC1, MAFK, NAB2, PAGR1, POLR3A, SMYD1, SMAD3, THAP1, BZW1, CLOCK, CPNE1, CCNT, CCNT2, KAT6A, KDM2A, MAPK3, NFIC, NFIX, PARK2, PTPN14, PTMA, PURB, TXNIP, TCF3, TBL1XR1, TLE4, ZBTB10, ZBTB34, ZBTB8A, ZNF276, ZNF391, ZNF426, ZNF429, ZNF585B, ZNF662, ZNF813.
Protein polyubiquitination	8	8.9 × 10^−3^	BCL2, TMEM189-UBE2V1, CUL3, KLHL42, PARK2, RNF138, RNF20, UBE2V1

### 3.5. Clinical Significance of the Identified miRNAs

We calculated the estimated ORs in the LNM+ samples. Among the RT-qPCR-validated miRNAs, statistically significant associations were found for the overexpressed miR-548ac (OR 3.29 95% confidence interval (CI) 1.33–8.12, *p* = 0.010), miR-4534 (OR 3.41 95% CI 1.20–9.63, *p* = 0.021), and miR-92b-5p (OR 3.44 95% CI 1.10–10.80, *p* = 0.034). In addition, statistically significant associations were identified for the underexpressed miRNAs, including miR-487b (OR 0.25 95% CI 0.09–0.67, *p* = 0.005), miR-195 (OR 0.13 95% CI 0.03–0.59, *p* = 0.008), and miR-29b-2 (OR 0.34 95% CI 0.14–0.82, *p* = 0.016) (Table 3).

**Table 3:** Bivariate analysis of miRNAs associated with lymph node involvement in patients with early-stage cervical cancer treated with radical hysterectomy and bilateral pelvic lymphadenectomy.

microRNA	With Lymph Node Involvement 12 (48%)	Without Lymph Node Involvement 13 (52%)	OR (95% CI)	*p*
Overexpressed miRNAs				
miR-548ac	5.1 (4.8–5.5) ^†^	3.5 (3.1–3.9) ^†^	3.29(1.33–8.12)	0.010
miR-4534	5.0 (4.6–4.61) ^†^	4.1 (3.4–4.6) ^†^	3.41 (1.20–9.63)	0.021
miR-483-5p	5.5 (4.6–5.8) ^†^	4.4 (4.1–5.1) ^†^	2.40 (0.98–5.83)	0.053
miR-92b-5p	6.9 (6.5–7.2) ^†^	5.9 (5.5–6.6) ^†^	3.44 (1.10–10.80)	0.034
Underexpressed miRNAs				
miR-487b	4.0 (3.5–4.6) ^†^	5.8 (5.3–6.4) ^†^	0.25 (0.09–0.67)	0.005
miR-195	9.8 (9.3–10.0) ^†^	10.8 (10.2–11.2) ^†^	0.13 (0.03–0.59)	0.008
miR-29b-2-5p	3.9 (2.8–4.9) ^†^	5.1 (4.6–5.6) ^†^	0.34 (0.14–0.82)	0.016

Abbreviations: OR: odds ratio, miR: microRNA, CI: confidence interval. ^†^ Median (interquartile range).

## 4. Discussion

The analysis of global miRNA expression revealed a profile of 36 differentially expressed miRNAs in the two sample groups. Of note, this miRNA profile was able to correctly classify 91.6% of the LNM+ samples. We found statistically significant differences in the expression (over- or underexpression) of the seven miRNAs between patients with early-stage CC with or without LNM.

In 2018, Chen Q et al. analyzed the differential expression of 422 miRNAs in 145 patients diagnosed with early-stage CC (including 32 LNM+ patients and 113 LNM− patients) in The Cancer Genome Atlas (TCGA) database. They found that 75 miRNAs were differentially expressed between the 2 sample groups [36]. Interestingly, our results are consistent for five miRNAs (miR-548, miR-379, miR-337, miR-487b, and miR-376c) and several gene targets associated with lymph node involvement. The identification of the miRNA profile associated with the LNM+ status in patients with early-stage CC represents a diagnostic tool with relevant clinical application because it allows not only a better understanding of the biology of this disease but also the timely identification of patients who will require more aggressive treatment, closer monitoring, and possible detection of early relapse.

Among the miRNAs identified, miR-195 had an OR of 0.13 (*p* = 0.008) and was underexpressed in LNM+ patients. Overexpression of miR-195 is associated with cell migration, proliferation, and invasion in CC [37]. DU et al. found that miR-195 is involved in the cell cycle in the G1 to S transition, downregulating the expression of CCND2 and inhibiting cell proliferation; likewise, miR-195 decreases the expression of the transcription factor MYB, which is related to cell migration and invasion [37]. Li et al. showed that miRNA-195 is underexpressed in HeLa and SiHa CC cell lines (*p* < 0.0001) and is involved in the D1 signaling pathway; miRNA-195 underexpression also promotes cell proliferation and invasion [38]. These data corroborate those of the present study, according to which miR-195 underexpression was found in occult LNM+ patients. Likewise, Zhou et al. found that miR-195 was associated with LNM+ status (*p* = 0.009), an advanced clinical stage (*p* = 0.011), and greater cervical stroma involvement (*p* = 0.03) in patients with CC due to an association with Smad3 protein regulation, which is related to the migration and proliferation of malignant cells in CC as well as in other cancers such as esophageal and prostate cancer [39]. The underexpression of miR-487b was also strongly associated (OR 0.25, *p* = 0.005) with the LNM+ status in this study. Underexpression of miR-487b has been shown to be related to CC in some studies; however, the literature on this subject is limited, although studies have detected a relationship with the progression of tumors of the digestive tract and brain and of adenocarcinomas of the prostate and lung [40,41,42,43]. In a study by Hata et al., miRNA-487b was described as a negative regulator of metastasis by regulating the KRAS gene in colorectal cancer [44]. miR-29b-2-5p was found to be underexpressed in LNM+ patients (OR 0.34, *p* = 0.016). Kinoshita et al. described that the miR-29 family of miRNAs, including miR-29a, miR-29b, and miR-29c, was significantly underexpressed in tumor tissues compared to nontumorous tissues [45]. A meta-analysis by Qi et al. indicated that underexpression of the miRNA-29 family is associated with OS (hazard ratio (HR) 1.57 95% CI 1.18–2.08) and the disease-free period (HR 1.51, 95% CI 0.99–2.30) [46]. Additionally, the miRNA-29b family has been associated with the induction of apoptosis through the Mcl-1, Bcl-2, AKT-2, and p53 signaling pathways as well as with the inhibition of metastasis by the Mcl-1, MMP-2, SOCs-2, and GATA-3 pathways, among others [47]. Regarding its relationship with CC, miR-29b underexpression was directly associated with the expression of the E6 and E7 proteins in human papillomavirus (HPV)-infected patients, resulting in a modification of the cell cycle via the CDK6 pathway, which triggers malignant epithelial cells [48].

Regarding the overexpressed miRNAs, miR-483-5p was overexpressed in LNM+ patients (OR 2.40, *p* = 0.053). Nagamitsu et al. found that miRNA 483-5p was overexpressed in patients with CC compared to those without CC, with FC > 3.0 (*p* = 0.01) [49]. In the present study, miR-483-5p was overexpressed by 2.21-fold (*p* = 0.001) in metastatic lymph nodes. Likewise, Nishi also found that serum miR-483-5p was significantly overexpressed in 40 patients with CC compared to 20 controls [50]. miR-4534 overexpression was strongly associated with LNM in patients with CC (OR 3.41, *p* = 0.021). No studies on the relationship between this miRNA and CC have been published; however, a relationship with prostate cancer was found. A study by Nip et al. reported that miRNA-4534 was overexpressed in a group of patients with prostate cancer. Likewise, the expression of this miRNA exerts an oncogenic effect by downregulating the PTEN suppressor gene. PTEN is a critical tumor suppressor gene related to survival, proliferation, cell migration, and angiogenesis through the PI3/Akt pathway [51]. Finally, another overexpressed miRNA associated with LNM+ status was miR-548ac, which was overexpressed in LNM+ patients (OR 3.29, *p* = 0.010).

The strengths of this study are that our findings were validated by RT-qPCR and were based on optimal results. A weakness of this study is that it was performed on a small number of samples; thus, further validation is required. Some of the miRNAs found in our study are associated with the development, proliferation, and migration of malignant cells in CC as well as other malignant cancers. Prospective studies with a greater number of individuals are essential to evaluate this profile as a possible biomarker related to early-stage occult LNM. The results could help identify individuals at high risk of nodal disease who are candidates for chemotherapy (CT)/radiotherapy (RT) instead of surgery, which carries a high risk of being incomplete because of nodal disease.

## 5. Conclusions

A seven-miRNA signature associated with LNM+ status was determined in patients with early-stage CC who were treated with RH and BPL. This signature exhibited prognostic value for identifying early-stage CC patients with LNM+ status. These findings may help detect lymph node micrometastases that cannot be observed in imaging studies.

## Data Availability

Data can be made available by direct request to the corresponding author.

## Supplementary Materials

The following materials are available online at https://www.mdpi.com/article/10.3390/curroncol29010023/s1. Supplementary File S1: Microarray data: contains the expression matrix of the normalized microarray data. DE miRNAs: contains the expression values of diferentially expressed miRNAs. qPCR Data: contains the raw data from the qPCR experiments and the calculations made to determine the fold change values. Target predicted: contains the list of predicted molecular targets for each miRNA. Keeg Pathways: contains the list of all keg pathways identified. Interaction networks: contains the data obtained for the construction of the interaction networks between the validated 7-miRNAs.
